# The Flavonoids Daidzein and Genistein Induce Wall‐Deficient Cell Formation in *Streptomyces coelicolor* Under Hyperosmotic Stress

**DOI:** 10.1111/1751-7915.70366

**Published:** 2026-06-05

**Authors:** Paula Valdés‐Chiara, Sergio Alonso‐Fernández, Angel Manteca, Gemma Fernández‐García

**Affiliations:** ^1^ Department of Functional Biology, Microbiology Area, IUOPA and ISPA, Faculty of Medicine Universidad de Oviedo Oviedo Spain; ^2^ Department of Physical and Analytical Chemistry, Faculty of Chemistry, ISPA University of Oviedo Oviedo Spain

**Keywords:** cell division, flavonoids, L‐forms, S‐cells, *Streptomyces*, wall‐deficient cells

## Abstract

Streptomycetes exhibit a complex multicellular life cycle, including programmed cell death and hyphal differentiation, and can generate extracellular vesicles and wall‐deficient cells such as S‐cells and L‐forms. These wall‐deficient morphotypes are increasingly recognised as functional bacterial states with emerging biotechnological relevance, yet the environmental signals that trigger their formation remain poorly defined. Here, we show that the flavonoids daidzein and genistein act as defined chemical inducers of wall‐deficient cell formation in *Streptomyces coelicolor* under hyperosmotic stress (0.64 M sucrose). Although the wild‐type strain fails to produce wall‐deficient cells in standard laboratory media under hyperosmotic conditions, robust formation of non‐dividing stress‐induced cells (S‐cells), along with a small proportion of dividing L‐forms, is observed in soya flour–mannitol medium (SFM) supplemented with high sucrose concentrations. We show that wall‐deficient cell formation can be induced by the addition of daidzein and genistein, the most abundant soybean flavonoids, to flavonoid‐free media containing high sucrose concentrations, at levels comparable to those found in SFM. These results identify flavonoids as defined chemical inducers of wall‐deficient morphogenesis in *Streptomyces*. More broadly, our findings suggest a link between plant secondary metabolites and bacterial morphological plasticity, with potential implications for plant–microbe interactions and microbial biotechnology.

## Graphical abstract

Plant‐derived isoflavones (daidzein and genistein) function as chemical signals that induce stress‐dependent formation of wall‐deficient cells (S‐cells) in *Streptomyces coelicolor*.

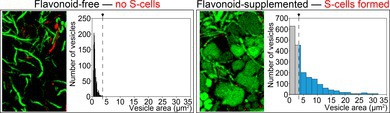

## 1 Introduction

Streptomycetes are filamentous actinobacteria of outstanding biotechnological relevance, responsible for the production of more than two‐thirds of the clinically used antibiotics as well as numerous anticancer, immunosuppressive and agroactive compounds (Barka et al. 2016; Van der Meij et al. 2017). Their complex multicellular life cycle includes programmed cell death, hyphal differentiation and sporulation, all of which rely on highly dynamic peptidoglycan (PG) remodelling and specialised cell division machineries (Flardh and Buttner 2009; McCormick and Flardh 2012; Yagüe et al. 2016). Beyond canonical septation, streptomycetes generate alternative morphotypes such as extracellular vesicles (EVs) and wall‐deficient cells, which are increasingly recognised as functional bacterial forms rather than developmental by‐products (Schrempf and Merling 2015; Meyer and Nodwell 2024). EVs are non‐replicative, membrane‐bound lipid bilayer particles naturally released by cells into the extracellular environment that carry a diverse cargo of proteins, lipids, nucleic acids and metabolites and mediate intercellular communication and functional exchange of biomolecules (Lotvall et al. 2014; Welsh, et al. 2024). Wall‐deficient cells comprise two major morphotypes: L‐forms, which are capable of autonomous replication and S‐cells, non‐replicative wall‐deficient cells specifically induced under hyperosmotic stress (Ramijan et al. 2018). S‐cells occupy a conceptual boundary between EVs and autonomous bacterial cells: although they resemble EVs in size and lack of a rigid cell wall and do not divide, S‐cells are metabolically active and contain the cellular machinery required to re‐establish filamentous growth, thereby representing true living cells rather than extracellular vesicles (Ramijan et al. 2018).

In diverse bacteria, L‐forms have been associated with antibiotic tolerance, phage resistance and persistent infections (Allan et al. 2009; Kawai et al. 2018), whereas in actinomycetes they have been proposed to contribute to stress adaptation, genome plasticity and alternative division strategies (Ramijan et al. 2018). Bacterial EVs have been exploited as versatile biotechnological tools for the delivery of bioactive molecules, vaccines and diagnostics, and for modulation of host–microbe and microbe–microbe interactions, owing to their natural capacity to encapsulate and transfer functional cargo (e.g., proteins and nucleic acids) across biological barriers (Moghaddam et al. 2025; Rima et al. 2025). Despite this emerging biotechnological relevance, the molecular mechanisms controlling EV, S‐cell and L‐form biogenesis in streptomycetes, and in bacteria in general, remain poorly understood. Recent work has demonstrated that wall‐deficient cell formation in *S. coelicolor* is not merely a passive biophysical consequence of osmoprotection but can be triggered by specific genetic alterations. The stomatin‐like protein StlP, involved in polar growth, was recently linked to the emergence of wall‐deficient cells under hyperosmotic stress in *S. coelicolor* (Zhong et al. 2025). In addition, the N‐acetyltransferase SCO0954, the D‐alanyl‐D‐alanine carboxypeptidase SCO4439, the GOLPH3‐like protein SCO4440 and the EngA GTPase SCO1758 have been shown to participate in peptidoglycan remodelling, and mutations in these proteins induce the formation of S‐cells and L‐forms in pre‐sporulating aerial hyphae (Alonso‐Fernandez et al. 2025). This morphogenetic transition occurs only under high osmotic stress (0.64 M sucrose) and not under lower osmotic conditions (0.3 M sucrose), despite the latter being sufficient to protect wall‐deficient cells from osmotic lysis, indicating that osmotic protection alone is not sufficient to induce the formation of these wall‐deficient cells (Alonso‐Fernandez et al. 2025).

The *S. coelicolor* wild‐type strain fails to generate wall‐deficient cells in standard glucose–yeast extract–malt extract (GYM) medium (Alonso‐Fernandez et al. 2025) and in other media such as L‐phase broth (LPB) when cultures are exposed to hyperosmotic conditions (0.64 M sucrose) (Ramijan et al. 2018). Here, we show that robust S‐cell formation, together with a small proportion of dividing L‐forms, occur in soya flour–mannitol (SFM) medium supplemented with high levels of sucrose (0.64 M), indicating that soybean‐derived components may act synergistically with osmotic stress to trigger wall‐deficient morphogenesis. We specifically identified daidzein and genistein as wall‐deficient cell elicitors. To the best of our knowledge, these flavonoids represent the first defined plant‐derived chemical signals shown to induce wall‐deficient cell formation in *S. coelicolor* under hyperosmotic stress.

## 2 Materials and Methods

### 2.1 Bacterial Strains and Culture Conditions

*Streptomyces coelicolor* A3(2) M145 was used throughout this study. Spores were obtained from cultures grown on soya flour–mannitol (SFM) agar plates for 7 days at 30°C following standard procedures for *Streptomyces* cultivation. Freshly harvested spores were used for all experiments.

Osmotic‐stress‐induced cell formation was analysed on solid GYM medium containing glucose (5 g L^−1^), yeast extract (4 g L^−1^), malt extract (5 g L^−1^), MgSO_4_·7H_2_O (0.5 g L^−1^) and agar (20 g L^−1^) (Novella et al. 1992). After autoclaving, the medium was supplemented with sterile K_2_HPO_4_ (0.5 g L^−1^). Soya flour mannitol (SFM) medium (Kieser 2000) was used as an alternative growth medium. Where indicated, media were supplemented with sucrose at final concentrations of 0.3 M or 0.64 M to impose osmotic stress.

Genistein (Extrasynthese, Genay, France) and daidzein (MedChemExpress, Monmouth Junction, NJ, USA) were added to the medium when required. Stock solutions of genistein and daidzein were prepared at 100 and 25 mg/mL, respectively in DMSO, sterilised by filtration and stored at −20°C. Flavonoids were added to the medium after autoclaving, prior to plating, at a final volume of 1 mL per litre of culture medium, with appropriate dilutions. Control media were prepared by adding an equivalent volume of DMSO. For plate‐based assays, 25 mL of medium were dispensed into 90 mm three‐vent Petri dishes. Plates were overlaid with sterile cellophane membranes and inoculated with approximately 3 × 10^7^ freshly harvested spores. Cultures were incubated at 30°C for the times indicated in each experiment.

### 2.2 Cell Morphology and Viability

Cell morphology and viability were analysed using propidium iodide (PI) and SYTO9 from the LIVE/DEAD BacLight Bacterial Viability Kit (Invitrogen). Staining solutions were prepared in a sucrose solution (103 g L^−1^) to prevent osmotic lysis of wall‐deficient cells. Samples were examined using a Leica TCS‐SP8 confocal laser‐scanning microscope. Fluorescence excitation wavelengths of 488 and 568 nm were used, and emission signals were collected at 530 nm (green channel) and 640 nm (red channel) (Manteca et al. 2008). Images were recorded and processed using standard confocal microscopy procedures.

### 2.3 Time‐Lapse Imaging

Time‐lapse microscopy was performed to analyse the dynamics of wall‐deficient cell formation and division. Cultures grown on cellophane membranes for 48 h were transferred to fresh medium containing SYTO9 (0.5 μmol L^−1^) and the membrane dye FM5‐95 (3.95 μg mL^−1^). Cellophane sheets carrying the mycelium were excised and placed onto fresh stained medium and then inverted onto coated μ‐dishes (Ibidi GmbH). Samples were incubated at 30°C during imaging.

Time‐lapse images were acquired using a Leica TCS‐SP8 confocal microscope every 13 min over a period of 17 h. Fluorescence excitation wavelengths of 488 and 522 nm were used, with emission signals collected at 530 nm (SYTO9) and 782 nm (FM5‐95). The microscope stage and chamber were equilibrated for approximately 3 h before imaging to minimise focal drift.

### 2.4 Membrane and Cell‐Wall Staining

Cell wall visualisation was performed using wheat germ agglutinin conjugated with Alexa Fluor 488 (WGA‐Alexa488; Invitrogen), which binds N‐acetylglucosamine and N‐acetylmuramic acid residues present in peptidoglycan. Hyphae were gently scraped from cellophane membranes and processed as described previously (Schwedock et al. 1997). Cells were fixed for 15 min at room temperature in phosphate‐buffered saline (PBS) containing 2.8% paraformaldehyde and 0.0045% glutaraldehyde. PBS consisted of 0.14 M NaCl, 2.6 mM KCl, 1.8 mM KH_2_PO_4_ and 10 mM Na_2_HPO_4_ and was supplemented with 103 g L^−1^ sucrose. Following fixation, samples were washed twice with PBS and briefly treated (1 min) with lysozyme (2 mg mL^−1^) prepared in glucose–Tris–EDTA buffer. This mild treatment facilitates WGA access to N‐acetylglucosamine residues in the cell wall (Biari et al. 2019). Cells were then washed again with PBS and blocked with 2% bovine serum albumin (BSA) in PBS for 5 min. WGA‐Alexa488 was added at a final concentration of 100 μg mL^−1^ in PBS containing 2% BSA, and samples were incubated for 3 h at room temperature. After staining, samples were washed several times with PBS. The membrane dye FM5‐95 (Thermo Fisher Scientific) was subsequently added at a final concentration of 3.95 μg mL^−1^.

Samples were examined using confocal laser‐scanning microscopy with excitation at 498 nm and emission collected at 511–559 nm for WGA‐Alexa488 (green channel) and 700–795 nm for FM5‐95 (red channel).

### 2.5 Image Analysis and Quantification

The size and abundance of osmotic‐stress‐induced cells were quantified from confocal images stained with PI and SYTO9. Image analysis was performed using the Fiji software platform. Cell detection was performed using a custom Fiji macro incorporating the StarDist segmentation plugin (Schmidt et al. 2018). For each detected structure, parameters including area, perimeter, circularity, Feret diameter, minimum Feret diameter, aspect ratio, roundness and solidity were calculated. A circularity threshold of 0.8 was applied (1 representing a perfect sphere).

Control images of *S. coelicolor* grown under non‐inducing conditions were used to determine the maximum vesicle area associated with normal hyphal structures. Based on these measurements, a threshold area of 3.6 μm^2^ was defined, as previously reported (Alonso‐Fernandez et al. 2025). Structures exceeding this value were classified as osmotic‐stress‐induced cells. At least 100 vesicles were quantified from images derived from three independent biological replicates.

Cell abundance was calculated as the proportion of vesicle‐associated stained area relative to the total stained area within each image. Total stained area was determined by measuring pixels above background intensity and converting this value to μm^2^. Background levels were estimated from regions lacking hyphae or vesicles.

### 2.6 Statistical Analyses

Differences in S‐cell area and abundance were analysed using the non‐parametric Mann–Whitney *U* test, as the data did not follow a normal distribution. *p*‐values < 0.05, < 0.01 and < 0.001 were considered statistically significant.

## 3 Results

### 3.1 *Streptomyces coelicolor* Forms S‐Cells and a Small Proportion of L‐Forms Under Hyperosmotic Stress in SFM Medium

**FIGURE 1:**
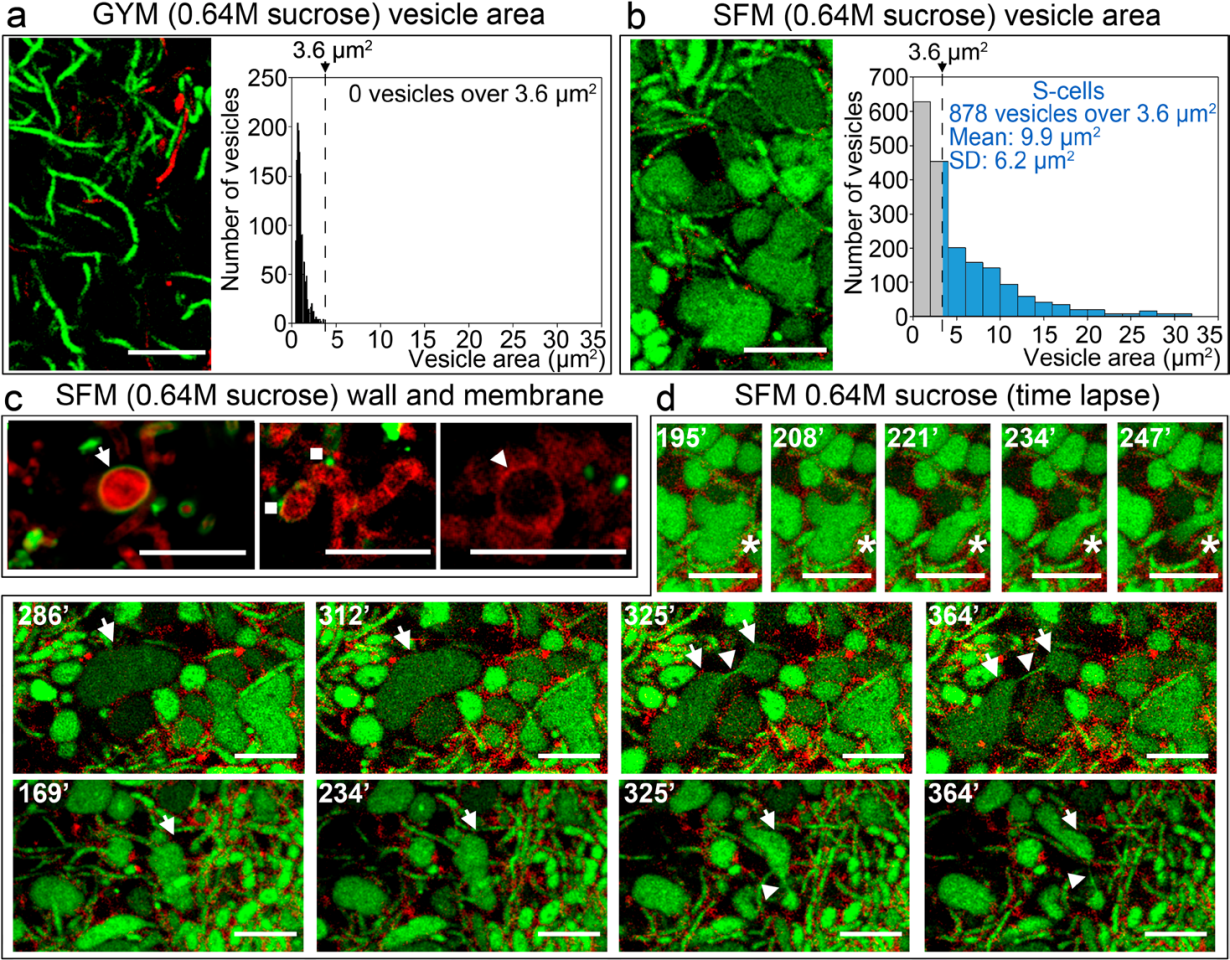
Formation of wall‐deficient cells under sucrose‐induced hyperosmotic stress in *Streptomyces coelicolor* grown on SFM medium supplemented with 0.64 M sucrose. (a, b) Confocal laser‐scanning fluorescence microscopy of hyphae stained with SYTO9 and PI (DNA stains) obtained from GYM or SFM media supplemented with 0.64 M sucrose. Histograms represent cell size (area): Grey bars correspond to the *S. coelicolor* wild‐type strain grown on GYM medium supplemented with 0.64 M sucrose (negative control, not producing wall‐deficient cells), used to establish the threshold area for wall‐deficient cell identification; blue bars represent cells with an area above the 3.6 μm^2^ threshold in cultures grown on SFM medium supplemented with 0.64 M sucrose. (c) Cell wall (Alexa Fluor 488‐WGA, green) and membrane (FM5‐95, red) staining. Arrows indicate thin cell walls, arrowheads indicate wall‐deficient cells, and squares indicate discrete cell wall patches. (d) Time‐lapse imaging showing the dynamics of wall‐deficient cell division in SFM medium supplemented with 0.64 M sucrose. Cultures were stained with SYTO9 (green, nucleic acid stain) and FM5‐95 (red, membrane stain). Images and time‐lapse recordings were obtained from 48 h cultures, once wall‐deficient cells had formed. Arrows indicate wall‐deficient cells undergoing division, arrowheads indicate transient membranous connections between dividing cells, and asterisks indicate moving and deforming cells. Time points (minutes) are indicated. Representative images from at least three biological replicates are shown in all cases. Scale bars: 8 μm.

As previously reported, the *S. coelicolor* wild‐type strain does not form S‐cells in GYM medium under high osmotic stress (0.64 M sucrose) (Alonso‐Fernandez et al. 2025). Under these conditions, only small hyphal bulges are detected by automated image analysis using Fiji (all below 3.6 μm^2^ in area; grey bars in the histogram in Figure 1a). In contrast, in SFM medium supplemented with 0.64 M sucrose, numerous round cells are produced above the 3.6 μm^2^ threshold (878 of 1904 detected structures), with a mean area of 9.9 ± 6.2 μm^2^ (blue bars in the histogram in Figure 1b). These cells are surrounded by membranes (FM5‐95, red staining) but lack a detectable cell wall (WGA, green staining), as indicated by arrowheads in Figure 1c, and therefore correspond to wall‐deficient S‐cells formed under hyperosmotic stress. As previously reported, some of these cells exhibit a very thin cell wall (arrows in Figure 1c), while others display well‐defined wall patches (squares in Figure 1c), which may correspond to sites of peptidoglycan degradation or synthesis (Alonso‐Fernandez et al. 2025).

A subset of wall‐deficient cells (9 out of 137 observed in the time‐lapse experiments, 6%; indicated by asterisks in Figure 1d; Movie S1) exhibits striking morphological deformation and dynamic movement. Within this population, a smaller subset (3 out of 137 cells, 2.2%; indicated by arrows in Figure 1d; Movie S1) is able to move and undergo division, as revealed by time‐lapse microscopy, and therefore represents L‐forms.

L‐forms establish transient membranous connections between dividing cells (arrowheads in Figure 1d), which, as discussed below, resemble midbody‐like structures described in eukaryotic cells (Hu et al. 2012) and have been previously reported during L‐form division in *Listeria monocytogenes* (Studer et al. 2016).

Under lower osmotic stress (0.3 M sucrose, a concentration sufficient to protect wall‐deficient cells from osmotic lysis) (Alonso‐Fernandez et al. 2025), only a small fraction of the structures quantified in SFM medium exceeded the 3.6 μm^2^ threshold (326 of 1634 detected structures), with a mean area of 4.6 ± 1.8 μm^2^ (Figure S1a). In GYM medium supplemented with 0.3 M sucrose, no structures exceeding the 3.6 μm^2^ threshold were observed, either in the presence or absence of flavonoids (Figure S1b–e). These results indicate that some component of the SFM medium promotes wall‐deficient cell formation; however, as previously reported for the *sco0954*, *sco4439*, *sco4440* and *sco1758* *S. coelicolor* mutants growing in GYM medium, high osmotic stress (0.64 M sucrose, but not 0.3 M sucrose, despite the latter being sufficient to prevent osmotic lysis) is also required to induce wall‐deficient morphogenesis (Alonso‐Fernandez et al. 2025).

### 3.2 The Flavonoids Daidzein and Genistein Induce S‐Cell Formation in *S. coelicolor* Grown on GYM Medium Under Hyperosmotic Stress

The selective formation of S‐cells in SFM but not in GYM medium suggested that undefined soybean‐derived components act synergistically with osmotic stress to trigger wall‐deficient morphogenesis. Soybean flour is particularly rich in the isoflavones daidzein and genistein, present at approximately 1.05 and 1.11 mg g^−1^ dry weight, respectively (Hsu et al. 2010), corresponding to estimated concentrations of ~21 μg mL^−1^ daidzein and ~22.2 μg mL^−1^ genistein in SFM medium. We therefore tested whether these flavonoids could induce S‐cell formation when added to flavonoid‐free GYM medium.

**FIGURE 2:**
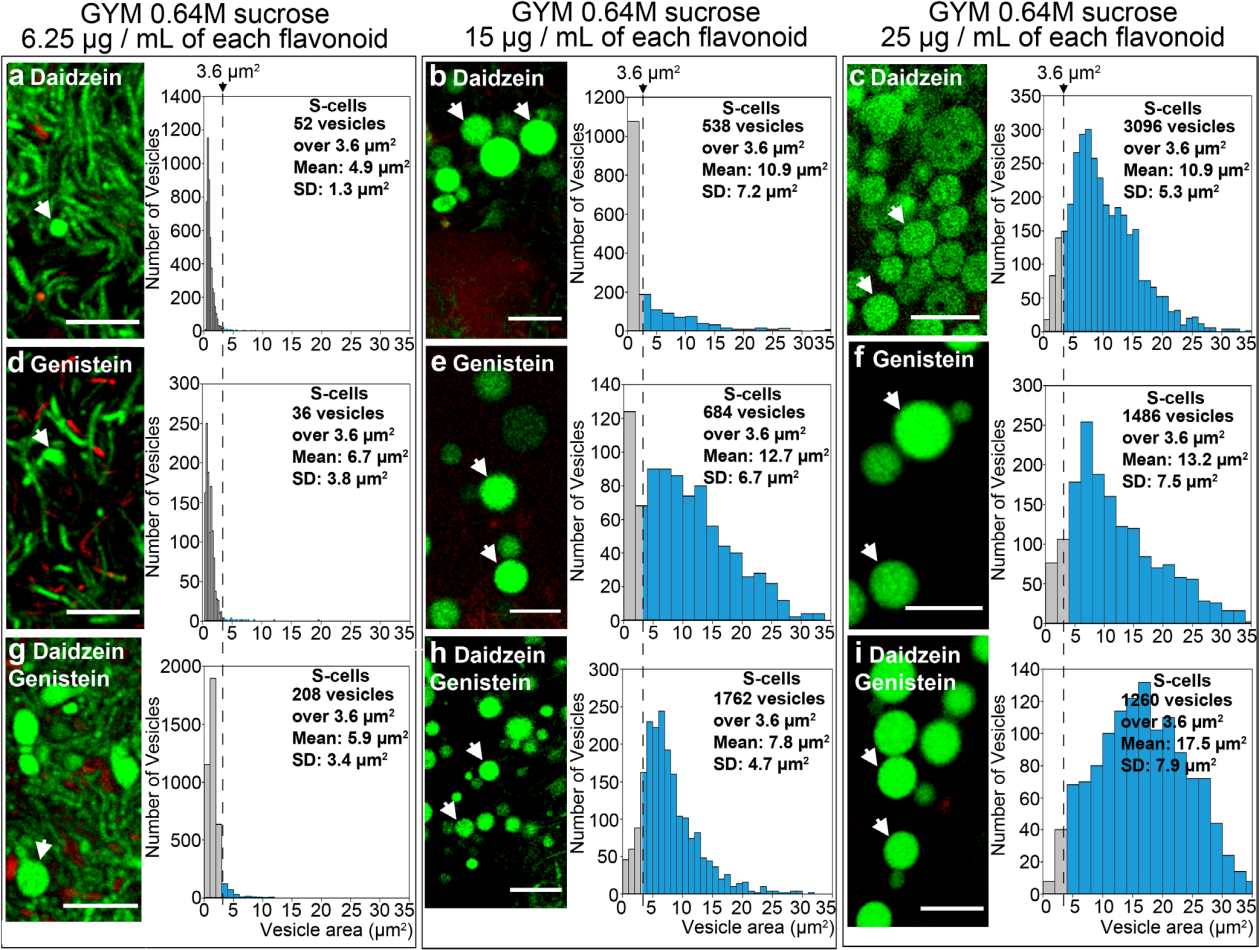
Formation of wall‐deficient cells under sucrose‐induced hyperosmotic stress in *S. coelicolor* grown on GYM medium supplemented with 0.64 M sucrose and different concentrations of daidzein and genistein. Images and histograms are presented as in Figure 1. Arrows indicate wall‐deficient cells. Scale bars represent 8 μm.

Low concentrations of either daidzein or genistein (6.25 μg mL^−1^) induced low but detectable levels of round cell formation (Figure 2a,d), which were enhanced when both flavonoids were applied together (Figure 2g; mean area 5.9 ± 3.4 μm^2^). Higher concentrations (15 μg mL^−1^) markedly increased this kind of round cell formation (Figure 2b,e,h). The highest levels of round cell formation were observed at 25 μg mL^−1^, a concentration comparable to that naturally present in the soya flour used to prepare SFM medium. Under these conditions, large S‐cells were produced (Figure 2c,f,i), with a mean area of 17.6 ± 7.9 μm^2^ when both flavonoids were combined (Figure 2i).

**FIGURE 3:**
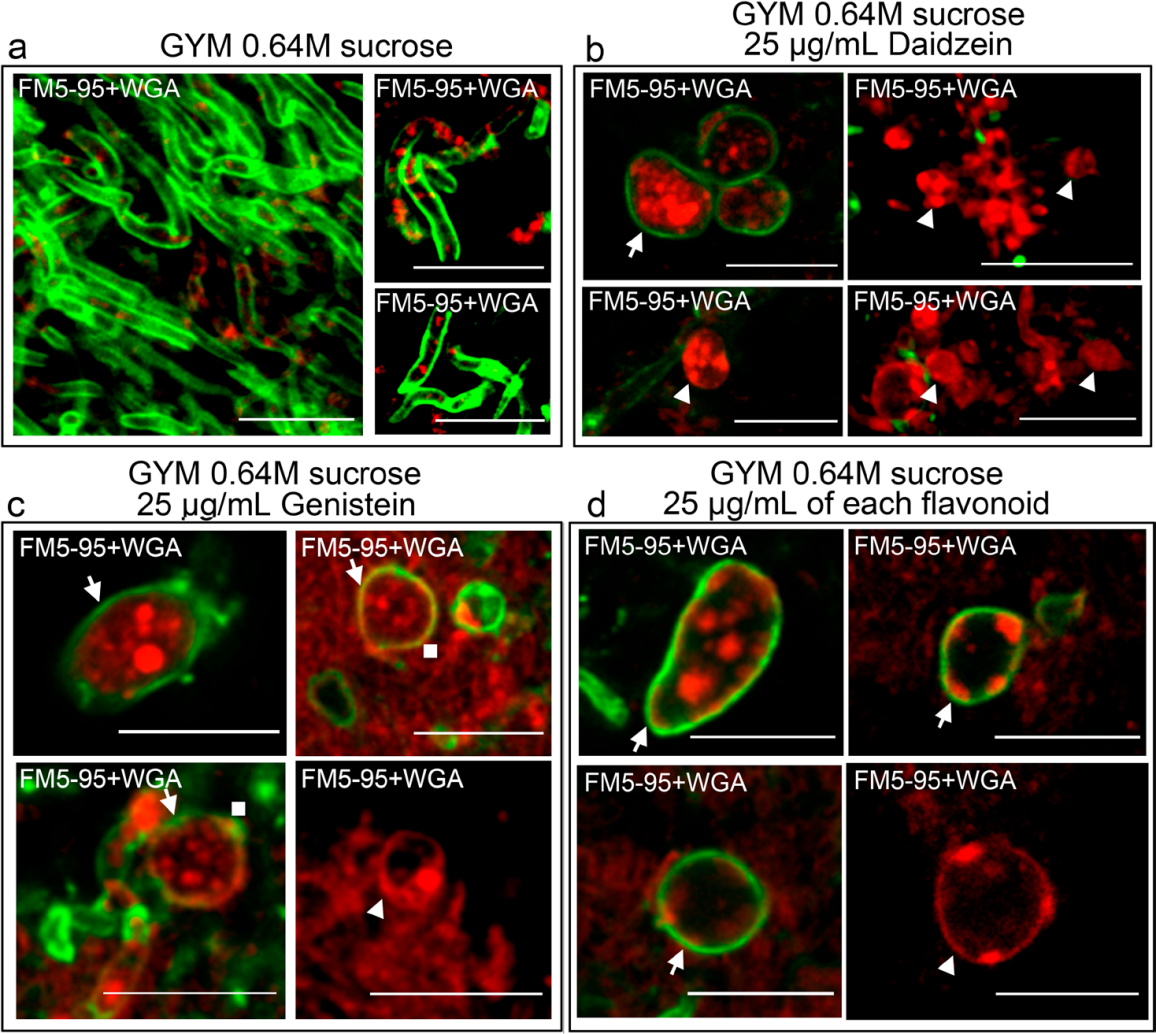
Cells formed under sucrose‐induced hyperosmotic stress and flavonoid supplementation in *Streptomyces coelicolor* grown on GYM medium lack a cell wall. Confocal laser‐scanning microscopy images of cultures grown on GYM medium supplemented with 0.64 M sucrose and stained with FM5‐95 (red, membrane stain) and Alexa Fluor 488‐WGA (green, cell wall stain). (a) *Streptomyces coelicolor* grown on GYM medium supplemented with 0.64 M sucrose but without flavonoids (negative control). (b) *S. coelicolor* grown on GYM medium supplemented with 0.64 M sucrose and 25 μg mL^−1^ daidzein. (c) *S. coelicolor* grown on GYM medium supplemented with 0.64 M sucrose and 25 μg mL^−1^ genistein. (d) *S. coelicolor* grown on GYM medium supplemented with 0.64 M sucrose, 25 μg mL^−1^ daidzein and 25 μg mL^−1^ genistein. Images correspond to the merged FM5‐95 and WGA channels. Arrowheads indicate cells without detectable cell walls; squares denote cells with thin cell walls; arrows indicate cells with thick peptidoglycan walls. Representative images from at least three biological replicates are shown. Scale bars represent 8 μm.

Cells induced by flavonoids under hyperosmotic conditions (0.64 M sucrose) in GYM cultures lack a cell wall and therefore correspond to S‐cells (Figure 3). As expected, no large, distinct round cells were detected in *S. coelicolor* grown without flavonoids (Figure 3a). In contrast, all cultures grown on GYM medium supplemented with 0.64 M sucrose and flavonoids produced large, distinct round cells with membranes (red) but without detectable cell walls (green) (arrowheads in Figure 3b–d). As previously described for the *sco0954*, *sco4439*, *sco4440* and *sco1758* mutants of *S. coelicolor* grown on GYM medium supplemented with 0.64 M sucrose (Alonso‐Fernandez et al. 2025), we also observed cells with thick cell walls (arrows in Figure 3b–d) and discrete peptidoglycan patches (squares in Figure 3b–d), which may represent remnants of the original cell wall or sites of peptidoglycan regeneration. Some vesicles displayed increased internal FM5‐95 staining, a characteristic feature of L‐forms (Siddiqui et al. 2006; Briers et al. 2012; Mercier et al. 2012).

### 3.3 S‐Cell Size and Abundance Correlate With Flavonoid Concentration

**FIGURE 4:**
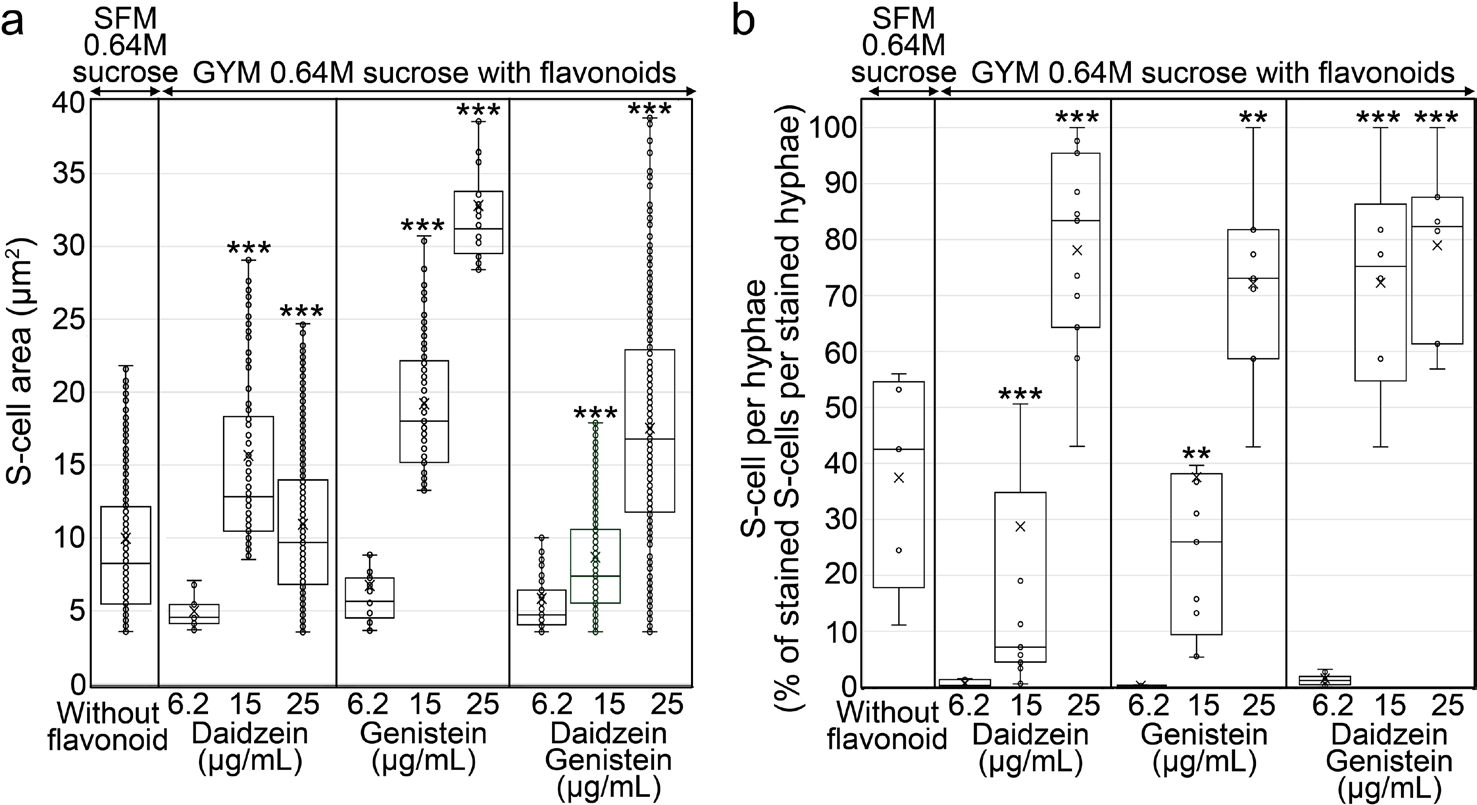
Area and abundance of S‐cells formed under sucrose‐induced hyperosmotic stress in *Streptomyces coelicolor* grown on SFM without and on GYM medium supplemented with daidzein and genistein. (a) Osmotic‐stress‐induced cell area. (b) Osmotic‐stress‐induced cell abundance. Box‐and‐whisker elements: Centreline, median; box limits, upper and lower quartiles; whiskers, 1.5× interquartile range. Asterisks indicate statistically significant differences compared with the lowest flavonoid concentration (6.2 μg mL^−1^): *p < 0.05, **p < 0.01, ***p < 0.001.

Quantification of S‐cell size (cell area; Figure 4a) and abundance (percentage of stained S‐cells per stained hyphae; Figure 4b) revealed a clear dose‐dependent relationship between flavonoid concentration and both S‐cell size and abundance. Both S‐cell area and abundance showed significant increases (indicated by asterisks in Figure 4a,b) in GYM medium amended with 15 and 25 μg mL^−1^ flavonoids compared with cultures amended with 6.25 μg mL^−1^.

The most effective conditions for S‐cell induction were 25 μg mL^−1^ genistein or daidzein, applied either individually or in combination, producing S‐cells reaching areas of up to 35 μm^2^ (Figure 4a) and abundances approaching 100%, in which virtually all cells adopted the S‐cell morphology and no filamentous hyphae were observed (Figure 4b).

The sizes of S‐cells formed in SFM medium were comparable to those formed in GYM medium supplemented with 15 μg mL^−1^ daidzein or genistein, either individually or combined (Figure 4a). Similar S‐cell abundances were observed when daidzein or genistein were applied separately at 15 μg mL^−1^ (Figure 4b), whereas combined treatment with both flavonoids at 15 μg mL^−1^ resulted in complete conversion to S‐cells (approximately 100%; Figure 4b). Genistein at 25 μg mL^−1^, either alone or in combination with daidzein, strongly increased S‐cell size compared with cultures grown in SFM medium or cultures supplemented with 15 μg mL^−1^ flavonoids (Figure 4a). Moreover, both flavonoids at 25 μg mL^−1^ increased S‐cell production to nearly 100% (Figure 4b).

### 3.4 Effect of *sco0954*, *sco4439*, *sco4440* and *sco1758* Mutations on S‐Cell Formation in SFM Medium Under Hyperosmotic Stress

As introduced above, the N‐acetyltransferase SCO0954, the D‐alanyl‐D‐alanine carboxypeptidase SCO4439, the GOLPH3‐like protein SCO4440, and the EngA GTPase SCO1758 have been shown to participate in peptidoglycan remodelling, and mutations in the genes encoding these proteins induce the formation of S‐cells and L‐forms in pre‐sporulating aerial hyphae (Alonso‐Fernandez et al. 2025).

**FIGURE 5:**
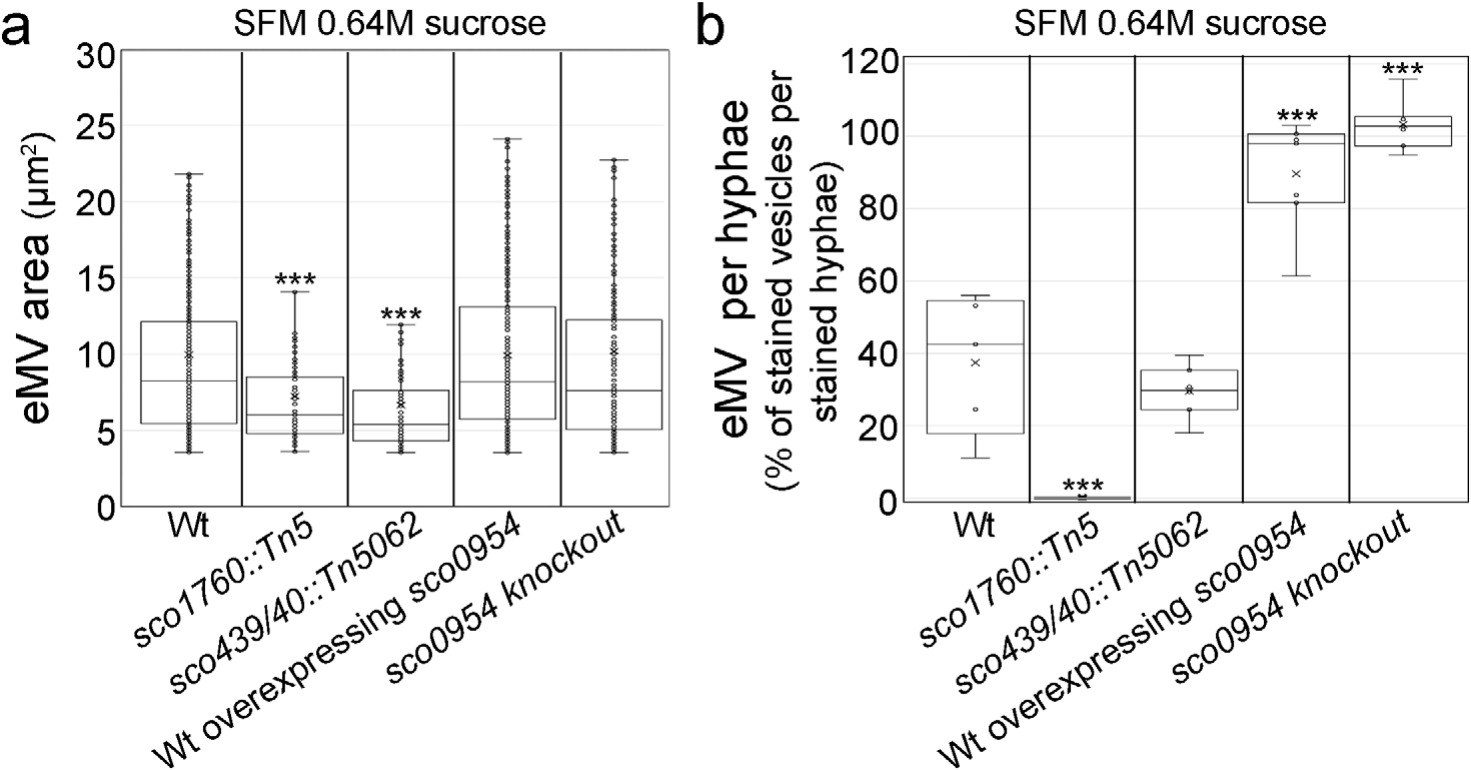
Differences in S‐cell size and abundance in the *Streptomyces coelicolor* wild‐type strain, the *sco4439/40::Tn5062*, *sco0954* knockout, and *sco1760::Tn5* mutants, and *S. coelicolor* overexpressing *sco0954* grown on SFM medium. (a) Osmotic‐stress‐induced cell area. (b) Osmotic‐stress‐induced cell abundance. Cultures were grown on SFM medium supplemented with 0.64 M sucrose. Asterisks indicate statistically significant differences compared with the wild‐type strain (*p* < 0.05, *p* < 0.01, *p* < 0.001; one, two or three asterisks respectively). Box‐and‐whisker plots show the median (centreline), interquartile range (box limits), and 1.5× interquartile range (whiskers).

Here, we observed that osmotic stress‐induced cells in SFM cultures supplemented with 0.64 M sucrose are significantly smaller in the *sco1760::Tn5* mutant, which affects *sco1758* and is complemented by *sco1758* (Alonso‐Fernandez et al. 2025), and the *sco4439/40::Tn5062* mutant, than in *S. coelicolor* or the other mutants (Figure 5a). Their abundance is markedly reduced in the *sco1760::Tn5* mutant, whereas it approaches 100% in the *sco0954* knockout mutant and in the wild‐type strain overexpressing *sco0954* (Figure 5b). These results suggest a potential interaction between flavonoid‐induced pathways and peptidoglycan‐associated pathways.

## 4 Discussion

Soybean‐derived isoflavones are abundant in environments associated with leguminous plants and display documented bioactivity against bacteria including antimicrobial effects and interactions with cellular membranes (Ahuja et al. 2012). More detailed studies have shown that flavonoids can modulate bacterial physiology by affecting membrane integrity, permeability and redox homeostasis (Cushnie and Lamb 2005; Daglia 2012; Farhadi et al. 2019). Beyond their antimicrobial activity, plant flavonoids are well‐established signalling molecules in plant–microbe interactions, acting as specific chemical signals that modulate bacterial gene expression, physiology and differentiation (Hassan and Mathesius 2012; Weston and Mathesius 2013). In legume–rhizobium symbioses, the isoflavones daidzein and genistein function as host‐derived signals that activate nodulation gene expression and trigger profound bacterial differentiation and host invasion at micromolar concentrations (Phillips and Kapulnik 1995; Subramanian et al. 2006). These paradigmatic systems demonstrate that flavonoids can act as environmental indicators of plant tissues, inducing alternative bacterial developmental programmes rather than merely exerting toxic effects.

Our findings show that daidzein and genistein are sufficient to induce S‐cell and L‐form formation in *S. coelicolor* in the absence of other soybean‐derived components, thereby identifying plant secondary metabolites as chemical inducers of bacterial morphogenesis under the conditions tested. The concentration‐dependent effects observed here indicate that flavonoids operate within a narrow physiological window (Figure 2), reflecting the complex chemical ecology of plant–actinomycete interactions in soil environments (Van der Meij et al. 2017). Flavonoids are actively released into the rhizosphere through root exudation, where they function as signalling molecules, for instance inducing nodulation in leguminous plants at nano‐ to micromolar concentrations. In contrast, antimicrobial effects are generally observed at higher micromolar levels (~50–200 μM), where flavonoids can disrupt membrane integrity and cellular homeostasis (Cushnie and Lamb 2005; Daglia 2012; Farhadi et al. 2019). Their spatial distribution is highly heterogeneous, and local accumulation at the root surface may result in higher effective concentrations experienced by associated microorganisms (Hassan and Mathesius 2012; Weston and Mathesius 2013). The concentrations tested here (15–25 μg/mL, ~60–100 μM) are comparable to those used in studies of flavonoid‐mediated bacterial responses and may reflect locally elevated levels in plant‐associated microenvironments such as the rhizosphere; however, their in situ relevance remains to be determined. Although the biomolecular mechanisms underlying flavonoid‐induced S‐cell formation remain unknown, recent work has shown that S‐cell and L‐form formation in *S. coelicolor* is modulated by proteins involved in polar growth, such as the stomatin‐like protein StlP (Zhong et al. 2025), as well as by peptidoglycan (PG)‐remodelling proteins (SCO0954, SCO4439, SCO4440 and SCO1758) that alter muropeptide composition and the redox and acetylation status of PG‐anchored methionine residues (Alonso‐Fernandez et al. 2025). Mutants in the *sco0954*, *sco4439*, *sco4440* and *sco1758* genes are able to form wall‐deficient cells in GYM medium under hyperosmotic stress in the absence of flavonoids (Alonso‐Fernandez et al. 2025) and respond differentially to flavonoids present in SFM medium (Figure 5), suggesting a potential interaction between flavonoid activity and peptidoglycan‐associated pathways, although the underlying mechanisms remain to be determined. Flavonoids are known to affect bacterial membrane fluidity and oxidative stress responses (Araya‐Cloutier et al. 2018; Farhadi et al. 2019), processes that are intimately linked to PG synthesis and remodelling. Consistently, both daidzein and genistein display documented antibacterial activity against a broad range of Gram‐positive and Gram‐negative bacteria, where they disrupt membrane integrity, alter permeability and perturb cell envelope structure, thereby indirectly compromising peptidoglycan homeostasis (Cushnie and Lamb 2005; Daglia 2012; Farhadi et al. 2019). Only two flavonoids (daidzein and genistein) were examined in this study. Although these compounds are representative of the major soybean‐derived isoflavones, it remains unclear whether the observed effects are specific to this class of molecules or reflect a more general response to membrane‐active phenolic compounds. Our results provide a plausible framework in which flavonoids may lower the physiological barrier to wall‐deficient morphogenesis under hyperosmotic stress, thereby facilitating this transition in *S. coelicolor* . It will also be important to determine whether similar responses occur in other streptomycete species and to further elucidate the underlying biochemical mechanisms.

The ecological roles of S‐cells in streptomycetes remain incompletely understood. Bacterial L‐forms have been experimentally shown to establish intimate associations with plant tissues and have been proposed as potential intracellular partners in plant systems (Allan et al. 2009). Moreover, naturally occurring wall‐less bacteria provide a compelling precedent for plant colonisation in the absence of a rigid cell wall: phytoplasmas (Mollicutes) are obligate plant pathogens that lack peptidoglycan and inhabit phloem sieve elements, where their small, pleomorphic cells traverse sieve pores and spread systemically within the host (Oshima et al. 2004; Namba 2008). By analogy, stress‐induced S‐cells and L‐forms in soil‐dwelling actinomycetes may represent transient morphotypes that could facilitate movement through plant‐associated microhabitats and potentially influence interactions with plant hosts, although this remains to be experimentally validated. Within this framework, flavonoids may contribute to the induction of wall‐deficient cells, potentially influencing bacterial colonisation of these niches.

Division of bacterial L‐forms occurs independently of the canonical FtsZ‐based divisome and relies on membrane‐driven mechanisms that are not yet fully understood and fundamentally differ from septum‐mediated cytokinesis (Leaver et al. 2009; Errington 2013; Mercier et al. 2014). The transient membranous bridges observed here between *Streptomyces* L‐forms (arrowheads in Figure 1c) resemble midbody‐like structures described during L‐form division in *L. monocytogenes* , suggesting that wall‐deficient bacteria may employ membrane‐mediated cytokinetic strategies that show conceptual similarities to certain aspects of eukaryotic division mechanisms (Hu et al. 2012; Studer et al. 2016).

## 5 Conclusion

S‐cells and L‐forms represent a complementary class of membrane‐based bacterial states that, unlike extracellular vesicles, retain metabolic activity and the capacity to revert to filamentous growth. Our results identify plant flavonoids as defined chemical inducers of wall‐deficient morphogenesis in bacteria, suggesting a link between plant secondary metabolism and bacterial cell envelope plasticity. The discovery that flavonoids can induce wall‐deficient cells in *Streptomyces* provides a chemically controllable strategy to generate these morphotypes. This may open new avenues for their exploitation in microbial biotechnology, for example as platforms for molecular delivery, vaccines and diagnostics in a manner analogous to current applications of bacterial extracellular vesicles (Guo et al. 2025; Rima et al. 2025). However, further work will be required to fully elucidate the molecular mechanisms underlying this process and to assess its practical applicability.

## Author Contributions

**Paula Valdés‐Chiara:** conceptualization, investigation, formal analysis, methodology, writing – original draft. **Sergio Alonso‐Fernández:** conceptualization, investigation, formal analysis, methodology, writing – original draft. **Angel Manteca:** conceptualization, investigation, funding acquisition, writing – original draft, methodology, writing – review and editing, supervision. **Gemma Fernández‐García:** conceptualization, investigation, methodology, writing – original draft, writing – review and editing, supervision.

## Funding

This study was supported by Agencia Estatal de Investigación (PID2021‐122911OB‐I00, PID2024‐156811OB‐I00) and the Agencia de Ciencia, Competitividad Empresarial e Innovación Asturiana (SEKUENS) (IDE/2024/000742). Paula Valdés‐Chiara was supported by a predoctoral grant from the Asociación Española Contra el Cáncer en Asturias (PRDAS245960VALD). Gemma Fernández‐García was supported by a postdoctoral grant from the Instituto de Investigación Sanitaria del Principado de Asturias (ITM25‐POS‐N2‐0334). 

## Conflicts of Interest

The authors declare no conflicts of interest.

## Supporting information


**Figure S1:** Absence of wall‐deficient cells under sucrose‐induced hyperosmotic stress in *Streptomyces coelicolor* grown on SFM medium supplemented with 0.3 M sucrose. Images correspond to confocal laser‐scanning fluorescence microscopy of hyphae stained with SYTO9 and PI (DNA stains). Histograms represent cell sizes (areas): grey bars correspond to the *S. coelicolor* wild‐type strain grown on GYM medium supplemented with 0.3 M sucrose (negative control, not producing wall‐deficient cells), used to establish the threshold area for wall‐deficient cell identification; blue bars represent wall‐deficient cells with an area above the 3.6 μm^2^ threshold (only present in small amounts in cultures grown on SFM medium supplemented with 0.3 M sucrose). (a) Culture on SFM medium supplemented with 0.3 M sucrose. (b) Culture on GYM medium supplemented with 0.3 M sucrose. (c) Culture on GYM medium supplemented with 0.3 M sucrose and 25 μg mL^−1^ daidzein. (d) Culture on GYM medium supplemented with 0.3 M sucrose and 25 μg mL^−1^ genistein. (e) Culture on GYM medium supplemented with 0.3 M sucrose, 25 μg mL^−1^ daidzein and 25 μg mL^−1^ genistein. Scale bars represent 8 μm.

**Movie S1:** Time lapse analysis of the *Streptomyces coelicolor* wild‐type strain growing on 0.64 M sucrose‐supplemented SFM medium. Time‐lapse imaging was initiated at 48 h, once osmotic induced cells had formed. The cultures were stained with SYTO‐9 (green, DNA stain) and FM5‐95 (red, membrane stain), with images acquired every 13 min. Scale bars represent 8 μm.

## Data Availability

The data that support the findings of this study are available from the corresponding author upon reasonable request.
