# Supplementary material for: The Flavonoids Daidzein and Genistein Induce Wall‐Deficient Cell Formation in Streptomyces coelicolor Under Hyperosmotic Stress
Source: Microb Biotechnol. 2026 Jun 5;19(6):e70366. doi: 10.1111/1751-7915.70366 (PMC13238533; doi:10.1111/1751-7915.70366)
Supplement: Supplementary file 1 — Figure S1: Absence of wall‐deficient cells under sucrose‐induced hyperosmotic stress in Streptomyces coelicolor grown on SFM medium supplemented with 0.3 M sucrose. Images correspond to confocal laser‐scanning fluorescence microscopy of hyphae stained with SYTO9 and PI (DNA stains). Histograms represent cell sizes (areas): grey bars correspond to the S. coelicolor wild‐type strain grown on GYM medium supplemented with 0.3 M sucrose (negative control, not producing wall‐deficient cells), used to establish the threshold area for wall‐deficient cell identification; blue bars represent wall‐deficient cells with an area above the 3.6 μm2 threshold (only present in small amounts in cultures grown on SFM medium supplemented with 0.3 M sucrose). (a) Culture on SFM medium supplemented with 0.3 M sucrose. (b) Culture on GYM medium supplemented with 0.3 M sucrose. (c) Culture on GYM medium supplemented with 0.3 M sucrose and 25 μg mL−1 daidzein. (d) Culture on GYM medium supplemented with 0.3 M sucrose and 25 μg mL−1 genistein. (e) Culture on GYM medium supplemented with 0.3 M sucrose, 25 μg mL−1 daidzein and 25 μg mL−1 genistein. Scale bars represent 8 μm. [file MBT2-19-e70366-s001.pdf]

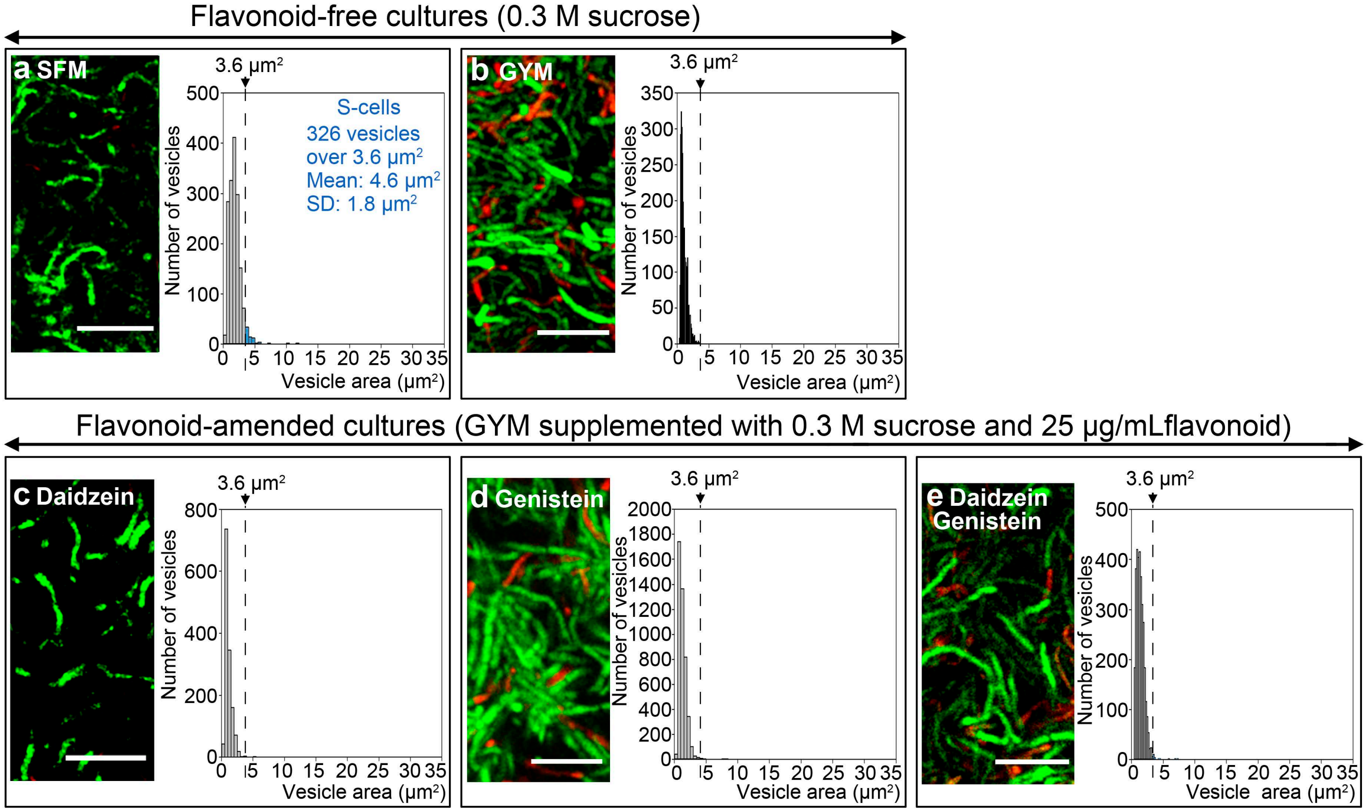

Figure S1. Absence of wall-deficient cells under sucrose-induced hyperosmotic stress in *S. coelicolor* grown on SFM medium supplemented with 0.3 M sucrose. Images correspond to confocal laser-scanning fluorescence microscopy of hyphae stained with SYTO9 and PI (DNA stains). Histograms represent cell sizes (areas): grey bars correspond to the *S. coelicolor* wild-type strain grown on GYM medium supplemented with 0.3 M sucrose (negative control, not producing wall-deficient cells), used to establish the threshold area for wall-deficient cell identification; blue bars represent wall-deficient cells with an area above the 3.6 µm² threshold (only present in small amounts in cultures grown on SFM medium supplemented with 0.3 M sucrose). (a) Culture on SFM medium supplemented with 0.3 M sucrose. (b) Culture on GYM medium supplemented with 0.3 M sucrose. (c) Culture on GYM medium supplemented with 0.3 M sucrose and 25 µg mL^{-1} daidzein. (d) Culture on GYM medium supplemented with 0.3 M sucrose and 25 µg mL^{-1} genistein. (e) Culture on GYM medium supplemented with 0.3 M sucrose, 25 µg mL^{-1} daidzein and 25 µg mL^{-1} genistein. Scale bars represent 8 µm.
